# Enhancing Low-Frequency Microwave Absorption Through Structural Polarization Modulation of MXenes

**DOI:** 10.1007/s40820-024-01437-x

**Published:** 2024-06-11

**Authors:** Bo Shan, Yang Wang, Xinyi Ji, Yi Huang

**Affiliations:** 1 College of Light Industry Science and Engineering, State Key Laboratory of Biobased Fiber Manufacturing Technology, Tianjin University of Science and Technology, Tianjin, 300457 People’s Republic of China; 2 National Institute for Advanced Materials, Tianjin Key Laboratory of Metal and Molecule Based Material Chemistry, Key Laboratory of Functional Polymer Materials, Collaborative Innovation Center of Chemical Science and Engineering (Tianjin), School of Materials Science and Engineering, Nankai University, Tianjin, 300350 People’s Republic of China

**Keywords:** Hierarchical structure, MXene, Microwave absorption, Low-frequency

## Abstract

Two-dimensional carbon-based materials have shown promising electromagnetic wave absorption capabilities in mid- and high-frequency ranges, but face challenges in low-frequency absorption due to limited control over polarization response mechanisms and ambiguous resonance behavior. In this study, we propose a novel approach to enhance absorption efficiency in aligned three-dimensional (3D) MXene/CNF (cellulose nanofibers) cavities by modifying polarization properties and manipulating resonance response in the 3D MXene architecture. This controlled polarization mechanism results in a significant shift of the main absorption region from the X-band to the S-band, leading to a remarkable reflection loss value of − 47.9 dB in the low-frequency range. Furthermore, our findings revealed the importance of the oriented electromagnetic coupling in influencing electromagnetic response and microwave absorption properties. The present study inspired us to develop a generic strategy for low-frequency tuned absorption in the absence of magnetic element participation, while orientation-induced polarization and the derived magnetic resonance coupling are the key controlling factors of the method.

**Supplementary Information:**

The online version contains supplementary material available at 10.1007/s40820-024-01437-x.

## Highlights

- MXene-based structural microwave-absorbing materials achieve an impressive reflection loss of − 47.9 dB in the low-frequency S-band, without the presence of magnetic materials and with low density.
- The elucidation of the mechanism behind the shift of the absorption band from high to low frequencies is attributed to the coupling of controlled multilevel polarization with induced electric field interactions.

**Supplementary Information:**

The online version contains supplementary material available at 10.1007/s40820-024-01437-x.

## Graphical abstract

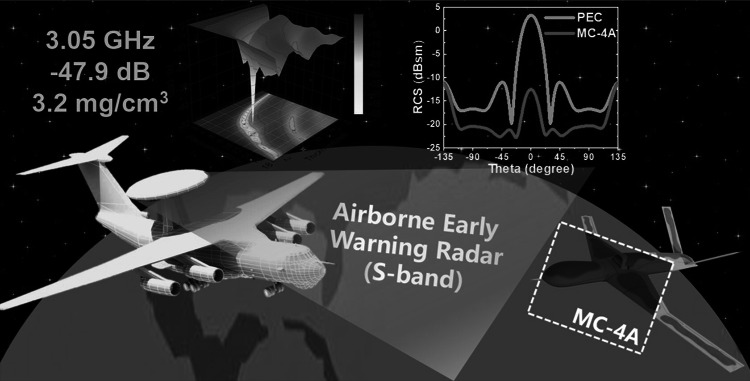

## Introduction

The management of electromagnetic waves is pivotal for the advancement of electromagnetic technologies and the development of sophisticated devices, holding promising prospects in defense, transportation, aerospace, and healthcare sectors [1–6]. Despite the strong electromagnetic wave (EMW) absorption and radar cross-section (RCS) reduction capabilities of certain two-dimensional (2D) carbon-based materials in mid- and high-frequency bands [7–11], the challenge of low-frequency absorption remains unresolved, particularly within the 2.5–3.7 GHz range critical for 5G and stealth defense technologies [12, 13].

MXene, emerging as prominent 2D nanomaterials with diverse types, has expanded the realm of 2D semiconductors and conductors [14]. MXene is derived from MAX phases during the acidic exfoliation process in which A-group elements are selectively removed. MAX phase is represented as M_*n*+1_AX_*n*_, *n* = 1–3, where M denotes transition metals, A stands for A-group elements, and X represents carbon (C) or nitrogen (N). MXene phase is represented as M_*n*+1_X_*n*_T_*x*_, where T_*x*_ denotes functional groups of –OH, –O, and –F. The exceptional electrical conductivity, large specific surface area and remarkable polarizability of MXene make it an ideal candidate for electromagnetic wave management [4, 15–18]. Typical Ti_3_CNT_*x*_ MXene self-assembled membranes provide absorption-dominated electromagnetic shielding, and the shielding effectiveness even surpassing metal materials [19]. However, excessive conductivity and disordered carrier migration induce a decrease in skinning depth and deteriorate the impedance matching at the material-air interface, thus triggering undesirable consequences in terms of EMW absorption. Therefore, most of the wave absorption studies of MXene mainly focus on the mid- and high-frequency bands where the skinning depth is lower, while in the low-frequency bands there is a lack of methodology and mechanism exploration for inducing electromagnetic coupling.

The strategy of resolving the low-frequency impedance mismatch by integrating magnetic materials such as magnetic cobalt, nickel, ferrite, alloys, and their composites to promote the absorption band move to the lower frequencies is very reasonable [5, 12, 20, 21]. Given that many magnetic materials response bands as low as MHz, the researchers sought to break Snoek’s limit by constructing a subtle anisotropic design and increasing the Curie temperature to bring the frequency response into the range that matches the incident electromagnetic wave [13, 22]. However, besides the anisotropic design, the magnetic response behavior is closely related to the critical grain size of the magnetic domains, and is mainly affected by short-range magnetic exchange interaction at lower than critical grain size. Hence it is vitally important to break the localized magnetic interactions and achieve long-range electromagnetic coupling [23]. Through the construction of abundant heterogeneous interfaces (0D/2D [24, 25], 1D/2D [21], 2D/2D [26–28], Core–shell [29]) and defect engineering, the interface polarization and dipole polarization can be effectively enhanced, thus allowing the magnetic field to pass through the incoherent heterogeneous interfaces as well as the grain boundaries with unequal charge distributions, and to radiate into the surrounding space [30–36]. For instance, Gu et al. reported a hierarchical magnetic microchain, which has a core–shell structure of two-dimensional Fe_*x*_Co_1−*x*_OOH nanosheets anchored vertically on the surface of a one-dimensional Co micro-chain. This long-range anisotropic magnetic microchain results in reflection loss (RL) below − 20 dB in C-band (4–8 GHz) [37]. Che et al. proposed an integrated magnetic resonance theory that includes macroscopic magnetic coupling, long-range magnetic diffraction, and nanoscale magnetic bridge connection. They constructed gradient magnetic domains to clarify the key role of long-range electromagnetic coupling [23]. Huang et al. exploited the electron spin interactions within the Fe_3_GeTe_2_ layer to generate room temperature ferromagnetism, further improving the multispectral compatible absorption performance by enhancing the magnetic loss capability of the material [38]. However, the magnetic materials that have to be added usually confront elevated densities, unpredictable low-frequency response, and environmentally unfriendly. So far, microwave absorption materials with properties including light weight and strong absorption are urgently needed to solve the electromagnetic hazard in lower-frequency bands such as S-band (2–4 GHz).

The three-dimensional (3D) EMW absorbing structures constructed by MXene have attracted a great deal of research attention [5, 15, 39–42], but the macrostructure-related discussion mainly focuses on improving the spatial transmission characteristics of electromagnetic waves, reducing the impedance matching at the interface and promoting multiple scattering [20, 22]. The possibility of 3D structures promoting low-frequency absorption has not been formally mentioned, mainly because the mechanism of structure-induced electromagnetic coupling is difficult to be clearly identified [38, 43]. Indeed, oriented 3D honeycombs can be readily used to tailor electromagnetic wave transmission channels with micrometer precision and effectively facilitate long-range orientation of large aspect ratio MXene. The orientation alignment of the MXene nanosheets will affect the carrier transport velocity or further induce polarization center separation, consequently altering their electromagnetic response properties [44–46]. This suggests that it is possible to control the response modes of MXene nanosheets to electromagnetic waves by adjusting the orientation of molecules or groups in the in-plane/out-of-plane direction. In addition, a phenomenon similar to magnetic resonance has been found to exist in partially non-magnetic two-dimensional materials under suitable electromagnetic field excitation [47, 48]. These magnetic resonances are usually accompanied by eddy currents, implying that even without the addition of a magnetic component there is a possibility of causing electromagnetic loss in which the magnetic effect intervenes.

In this work, we propose a tunable 3D cavities structure strategy to reshape the dipole polarization and interfacial polarization contribution of MXene, utilizing the transition-metal dangling bond sites, functional groups and a large number of heterogeneous surfaces as polarization centers. Due to unsaturated charges during the MXene exfoliation, strong metallicity and large density of states near the Fermi level, make it interesting to achieve long-range electromagnetic coupling in 3D MXene structure [47]. This strategy not only endowed MXene nanosheets with superior electromagnetic wave absorption properties, but also facilitated the realization of low-frequency tuning without the intervention of magnetic elements. The MXene/CNF composites (MC) demonstrated a satisfying original RL of up to − 52.6 dB in the X-band. Notably, by manipulation the response mode of the 3D cavities structure to the electromagnetic wave, the absorption band can be optimized and extended to the S-band, achieving an impressive RL value of − 47.9 dB. Our study proposes a general strategy for fabricating MXene-based materials for high-frequency and low-frequency EMW absorption, where orientation-induced polarization and derived magnetic resonance coupling are the key factors for achieving non-magnetic additive low-frequency absorption. This will open up new avenues for the development of low-frequency, lightweight, and eco-friendly EMW absorption materials.

## Experimental

### Materials

Lithium fluoride (LiF, 99.99%) and HCl (37%) were supplied by Aladdin (China), and MAX with a 400-mesh size and CNF were purchased from Kaixi Technology Ltd. (China) and NanoFC Ltd. (China), respectively. All materials were used as received.

### Synthesis of Few-Layer Ti_3_C_2_T_x_ MXene Sheets

Ti_3_C_2_T_x_ MXene was synthesized by etching of the parent Ti_3_AlC_2_ MAX phase, in a solution of concentrated hydrofluoric acid [49]. According to previous reports, due to the thermodynamic instability of the Ti–F bond in high-pH solutions, the F-terminal group can be easily replaced by hydroxyl groups in alkaline aqueous solutions to form water-soluble fluorine compounds [12, 44]. In view of this, we synthesized surface F-rich Ti_3_C_2_T_*x*_ nanosheets by leaving the exfoliated Ti_3_C_2_T_*x*_ nanosheets in pH = 5 etching solution at 40 °C for 36 h. For the solution preparation, 35 mL of 35 wt% hydrochloric acid, 2 g of lithium fluoride, and 10 mL of deionized water were thoroughly mixed using magnetic stirring in a fume hood to prevent the release of hazardous gases. Subsequently, 1 g of MAX (Kaixi Technology Ltd., with a 400-mesh size) was carefully added to the solution and transferred to a polytetrafluoroethylene (PTFE) plastic tube, which was sealed. The PTFE tube containing the solution was immersed in a water bath and the reaction was stirred for 24 h at 400 rpm and 35 °C. After the reaction, deionized water was added to the solution and the precipitate was collected by centrifugation at 3500 rpm. The obtained precipitate was re-dispersed in 50 mL of water and underwent multiple cycles of centrifugation (5 cycles) at 3500 rpm until the pH reached above 6. The solution was then agitated in an oscillator at high frequency for 30 min before another round of centrifugation at 3500 rpm to collect approximately 1/3 of the upper suspension volume as the MXene dispersion. The solution was shaken again and refrigerated at 2–8 °C, protected from light. The resulting MXene solution was shaken again and stored refrigerated at 2–8 °C, shielded from light.

### Preparation of 3D MC Cavities

MXene solutions with concentrations of 2.05–5 mg mL^−1^ were mixed with 2,2,6,6-tetramethylpiperidin-1-yloxy mediated oxidized nanofibrillated cellulose (CNF solution) to form mixed solutions in different ratios. The resulting mixture was homogenized for 5 min, followed by magnetic stirring for 30 min and sonication to ensure uniform distribution. The solution was then carefully poured into a rectangular copper mold fitted with a PTFE sealing liner, ensuring unidirectional heat transfer in the mold. The bottom of the copper mold was immersed in liquid nitrogen to act as a cold trap, with an immersion depth of 5 mm, allowing controlled growth of ice crystals from the bottom up at a rate of 0.04 mm s^−1^. Subsequently, the ice crystals were sublimated by vacuum for 36 h to produce the MC cavities. The obtained MC cavities were dried in a vacuum oven at 60 °C for 12 h. Based on the different ratios of 20, 30, and 40 wt% of MXene in the initial solution, the obtained aerogels were named MC-2, MC-3, and MC-4, respectively. Furthermore, the oriented aerogels were defined in terms of their opening and radial directions, referred to as A and R, respectively. Specifically, when the opening direction of an oriented aerogel was aligned with the direction of electromagnetic wave propagation, the sample was identified as MC-A. Conversely, aerogels in which the opening direction was perpendicular to the wave propagation direction were designated as MC-R.

### Characterizations

Field-emission scanning electron microscopy (FESEM, JEM7800F) was employed for determining the aerogel morphological features. Atomic force microscopy (AFM) was conducted on a DI Multimode V scanning probe microscope (Veeco Co. Ltd. U.S.) in the tapping mode. X-ray diffraction (XRD) and a Bruker advance at 40 kV/40 mA with Cu K radiation (*λ* = 0.154 nm) were employed to characterize the crystal structures of the sample. The zeta potential was tested by Malvern particle size & zeta potential analyzer (Nano S90, UK). To measure the RL and electromagnetic interference shielding effectiveness (EMI SE) performance of the aerogels, the prepared aerogels were cut into coaxial cylinders with an inner diameter of 3 mm, an outer diameter of 7 mm, and a thickness of 3 mm. The scattering parameters of the aerogel samples were measured by a vector network analyzer (PNA-N5222B) in the frequency range of 2–18 GHz.

*RCS simulation* The RCS of a metal plate covered and uncovered with MC absorbing material was simulated using CST Studio Suite 2018. The metal plate was made of Perfect Electroconductivity (PEC) material with dimensions of 100 × 100 mm^2^ and an overall height of 1 mm. A sample covered with MC-4A as a stealthy functional layer of the metal plate was also observed. Due to its superior low-frequency stealth performance, the size of the MC-4A cover layer was 100 × 100 mm^2^, with a height set to 8.51 mm as the control height to maximize the reflection loss in the low-frequency S-band. Open boundary conditions were applied in all directions, with the azimuth angle determined by theta and phi in spherical coordinates. For the Gigahertz band, the monitoring wavelength in the modeling was 3.05 GHz, and a single-station RCS simulation was performed based on the electromagnetic parameters of MC-4A.

## Results and Discussion

### Construction of 3D MC Cavities

**Fig. 1:**
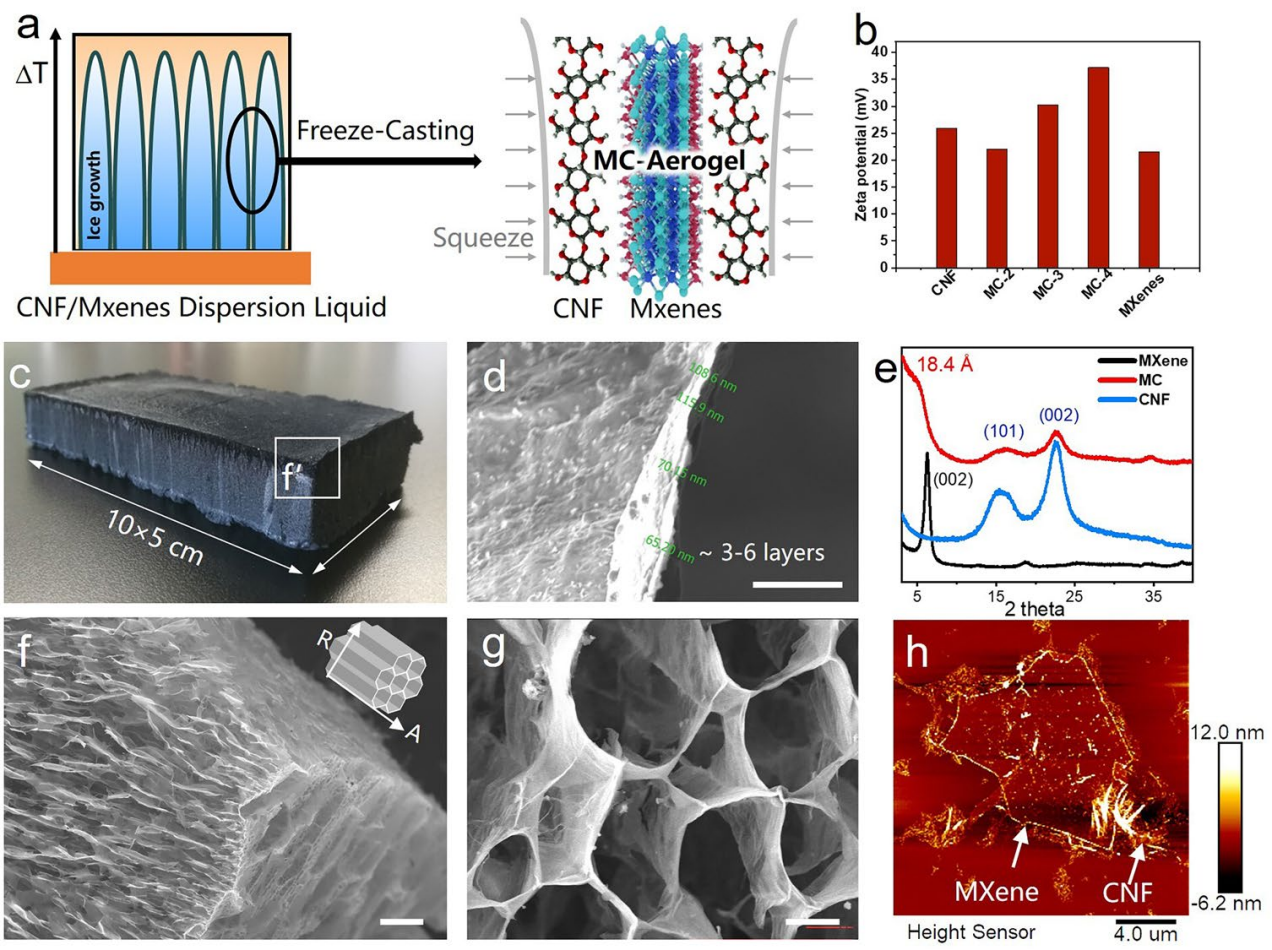
**a** Schematic diagram of the formation of 3D MC. **b** Zeta potentials of different sample dispersions. **c** Digital photograph of the MC, with the view position of SEM. **d, f, g** SEM images at different magnifications with bar scales of 1, 100, and 10 μm. **e** XRD spectra of samples. **h** AFM image of MC in dispersed liquid

To address the imperfect impedance matching at the MXene-air interface, a 3D porous cavity structure was designed and fabricated using the freeze-casting technique, as illustrated in Fig. 1a. Owing to the inadequate self-supporting strength of the MXene interface, we employed CNF known for their superior dispersibility and eco-friendly properties as an auxiliary template for constructing nanoscale 2D materials [50–52]. The Zeta potential displayed in Fig. 1b demonstrated that the combination of MXene and CNF resulted in a more stable dispersed state of the system. Subsequently, as ice crystals formed, MXene gradually concentrated in the suspension, while CNF and MXene separated from the liquid phase, forming a robust interface bond through hydrogen bonding, dipole–dipole interactions, and CH-π interactions [53–55]. Upon sublimation of the ice crystals, the 3D-oriented structure aligned with the growth direction of the ice crystals was preserved.

As depicted in Fig. 1c, the MC aerogel blocks approximately 10 × 5 × 3 cm^3^. Samples with pore directions parallel to the direction of electromagnetic wave propagation were designated as “MC-A”, while those with pore orientation perpendicular to the direction of electromagnetic wave propagation were labeled as “MC-R”. 3D MC cavities with different densities (3.2, 5.3, and 9 mg cm^−3^) were, respectively, named MC-2, MC-3, and MC-4. XRD patterns illustrated in Fig. 1e indicated the semi-crystalline peaks of β-cellulose at 2*θ* = 23° [50, 52, 55], while the sharp characteristic peak at 2*θ* = 6.3° suggesting the preferential orientation of the exfoliated MXene nanosheets along the (002) crystal plane. In the MC, the 2*θ* angle of MXene shifted from 6.3° to 4.8°, with the corresponding MXene interlayer spacing increasing from 14 to 18.4 Å. This shift implied successful CNF insertion into the MXene interlayers, preventing van der Waals stacking [56]. The abundant intercalation of CNF in the MC facilitated the creation of more heterogeneous interfaces and induced rich dipole polarization [20, 43, 57]. The SEM image in Fig. 1f, g exhibited the highly oriented structure of the MC, showcasing significant variations in pore morphology under different observation angles. In addition, more SEM observations of Fig. S1 also show clear MXene wrinkled lamellar structures, consistent with previous morphological observations. Measurements indicated a pore width of approximately 30 μm and wall thickness ranging from 65–115 nm. Comparing this to the observed thickness of about 18 nm for the few-layer MXene/CNF nanosheets in AFM (Fig. 1h), it was estimated that the pore walls of the MC consisted of approximately 3–6 layers of MXene/CNF stacking. The thin wall thickness relative to a single MXene sheet's lateral dimension promoted highly anisotropic assembly of MXene nanosheets, resulting in the overall orientation of functional groups and metal dangling bonds on the pore walls [58].

### Anisotropic Electromagnetic Response of the MC Cavities

**Fig. 2:**
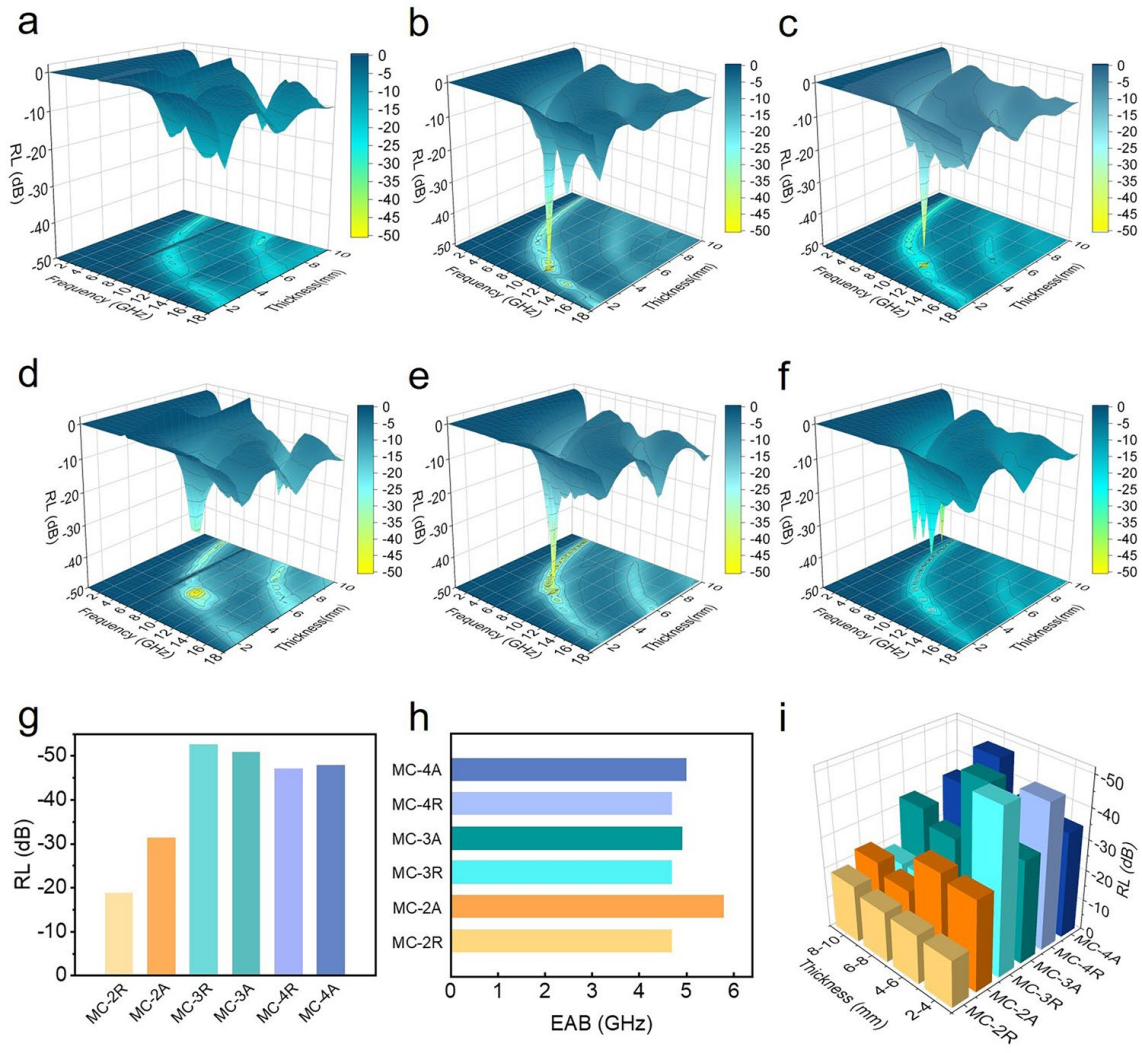
**a**–**f** 3D plot of the RL of **a** MC-2R, **b** MC-3R, **c** MC-4R,** d** MC-2A, **e** MC-3A and **f** MC-4A in the paraffin matrix. **g** Plots of different samples versus RL and EAB. **i** Columnar relationship diagram of thickness, sample, and RL

Figure 2 displays 3D plots of the RL values of the prepared materials in the frequency range of 2–18 GHz and in the absorber thickness range of 1–10 mm. The RL value was calculated by the transmission line theory [29, 41]:

1 $$ {\text{RL}} = 20\log \left| {\frac{{Z_{{{\text{in}}}} - Z_{0} }}{{Z_{{{\text{in}}}} + Z_{0} }}} \right| $$

2 $$ Z_{{{\text{in}}}} = \sqrt {\frac{{\mu_{r} }}{{\varepsilon_{r} }}} \cdot\tanh \left( {\frac{j \cdot 2\pi df}{c}} \right)\sqrt {\mu_{r} \varepsilon_{r} } $$

where ${Z}_{\text{in}}$ is the normalized input impedance of the sample; ${Z}_{0}$ is the impedance in free space; ${\varepsilon }_{r}$ and ${\mu }_{r}$ are the complex permittivity and complex permeability, respectively; *f* is the frequency; *c* is the speed of light in free space; and *d* is the thickness of the absorber. Figure 2 shows the 3D plots of the RL curves for different samples, and the statistical relationship between the maximum absorption intensity and the effective bandwidth is given in Fig. 2g, h. It is evident that the material’s maximum absorption intensity significantly increases with higher MXene content, reaching − 52.6 dB in MC-3A. On the other hand, the absorption bandwidth variation differed from the RL, with a maximum effective absorption bandwidth of 5.8 GHz achieved in MC-2R despite lower MXene content. This behavior was related to the dual-band absorption characteristics, which is similar to the 3D continuous cross-linked graphene honeycomb [59–62]. The multiple resonance losses associated with the increased skin depth of MC-2 hinted at the potential for further expansion of the effective bandwidth of MC in theory [19, 20, 40, 43, 57, 63]. Figure 2i illustrates the impact of different material thicknesses on absorption intensity, revealing a substantial increase in absorption intensity with matching thickness. This phenomenon could be elucidated by the free electron theory and effective medium theory in the next section [64].

Generally, absorption performance correlates with both the interface's impedance matching and its loss performance. To prepare suitable absorbing materials, it is necessary to coordinate both aspects simultaneously. The coefficient of interface impedance matching can be evaluated by calculating the parameter $\left|{Z}_{\text{in}}/{Z}_{0}\right|$, which is related to the impedance of air [65, 66]:

3 $$\left|{Z}_{\text{in}}/{Z}_{0}\right|=\sqrt{\frac{{\mu }_{r}}{{\varepsilon }_{r}}}$$

**Fig. 3:**
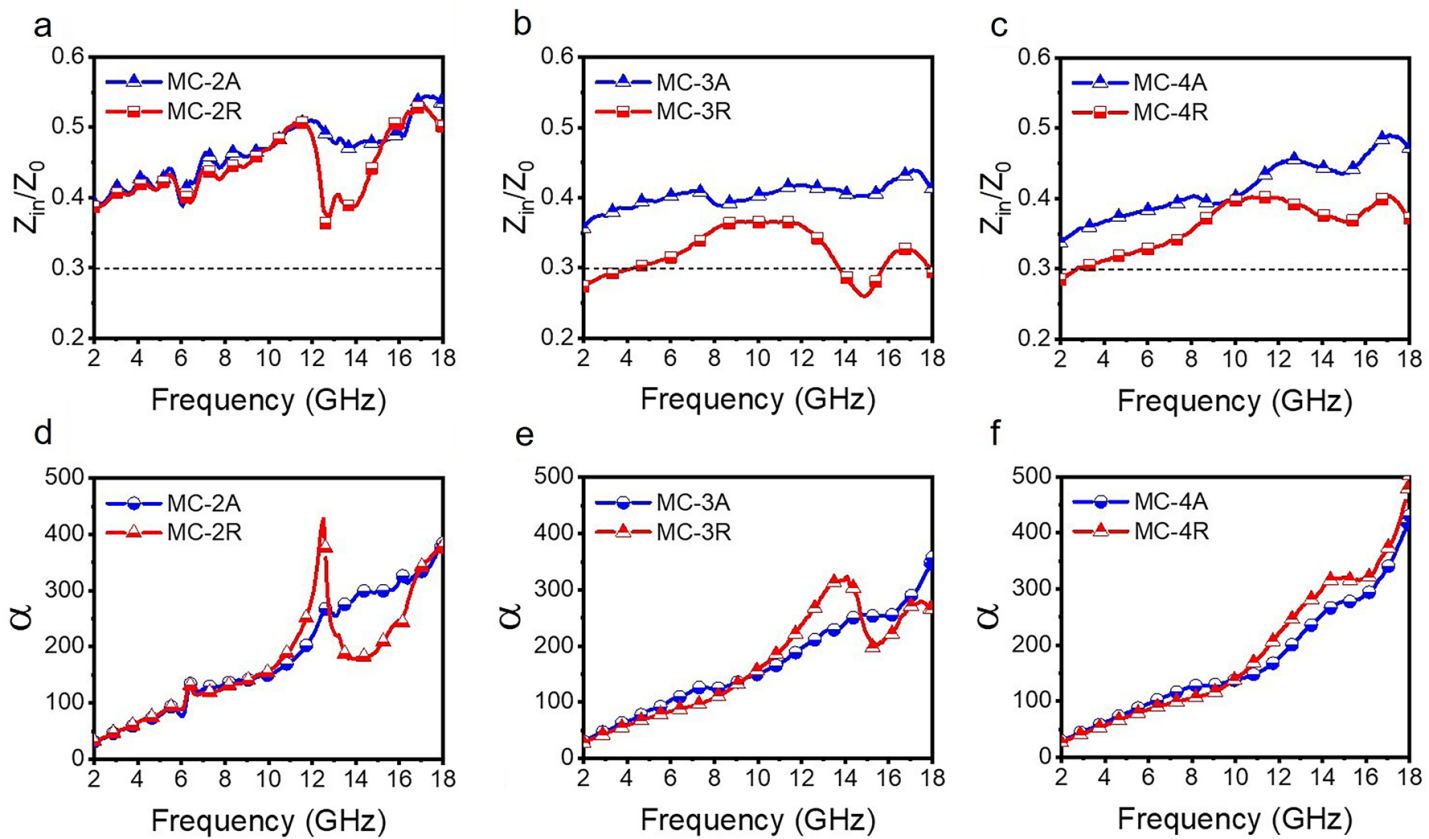
**a**–**c** Normalized electromagnetic wave impedance of the MC cavities in axial and **d**–**f** the radial direction and attenuation constant-frequency curve

Figure 3a–c depicts the impedance matching relationship of the MC. As the density increases, the impedance matching of the material interface gradually decreases. The “MC-A” series samples exhibit better impedance matching performance due to their lower dielectric constants. Furthermore, all samples have $\left|{Z}_{\text{in}}/{Z}_{0}\right|$> 0.3, which falls within the appropriate impedance matching range for the electromagnetic wave absorbing materials [67]. Although impedance matching is a prerequisite for introducing electromagnetic waves into the interior, the absorption loss ultimately determines the magnitude of attenuation performance. The attenuation loss coefficient α is usually calculated as follows [68]:

4 $$ \alpha = \frac{\sqrt 2 \pi f}{c} \times \sqrt {\left( {\mu^{\prime \prime } \varepsilon^{\prime \prime } - \mu^{\prime } \varepsilon^{\prime } } \right) + \sqrt {\left( {\mu^{\prime \prime } \varepsilon^{\prime \prime } - \mu^{\prime } \varepsilon^{\prime } } \right)^{2} + \left( {\mu^{\prime } \varepsilon^{\prime \prime } + \mu^{\prime \prime } \varepsilon^{\prime } } \right)^{2} } } $$

Figure 3d–f shows that there is not a significant increase in α with the increase of MXene content. However, a strong resonance peak is observed in MC-2R, while in MC-3R and MC-4R, the resonance peak weakens and shifts toward higher frequencies. This observation is supported by the Tanδ in Fig. S4. We will discuss this resonance in the next section.

### Low-Frequency Absorption of MC Cavities

**Fig. 4:**
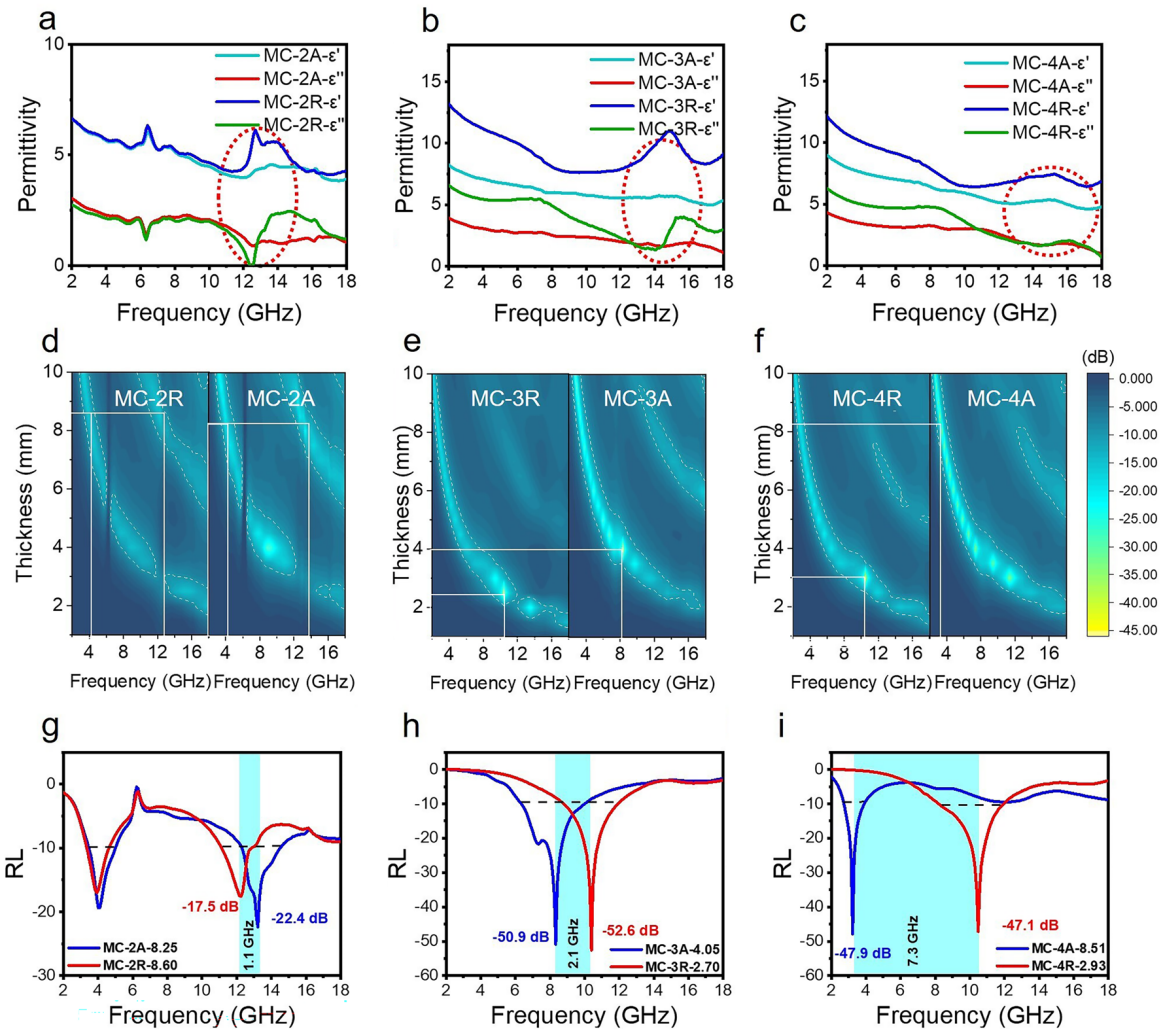
**a**–**c** Complex permittivity for MC-2, MC-3, and MC-4 along the axial and the radial direction, respectively. **d**–**f** Electromagnetic wave reflection loss and the corresponding 2D contour plots for MC-2, MC-3, and MC-4 along the axial and the radial direction, respectively, and the white dotted line is the boundary of − 10 dB. **g**–**i** RL versus frequency of MC-2, MC-3, and MC-4 at optimized thicknesses

For in-depth analysis, we measured electromagnetic parameters including relative complex permittivity and complex permeability in the frequency range of 2–18 GHz based on the coaxial line method. The real parts (*ε*′ and *μ*′) of the complex permittivity and complex permeability are associated with the storage of electric/magnetic energy, while the imaginary parts (*ε*″ and *μ*″) denote energy dissipation [64]. Figure 4 displays the dielectric constant and microwave absorption properties of the MC cavities. Figure 4a–c illustrates that the values of *ε*′ and *ε*″ increase with density throughout the test frequency range. Both *ε*′ and *ε*″ of MC-R are higher than those of MC-A. The MC-R series samples exhibit notable dielectric resonance phenomena, as mentioned earlier in loss coefficient *α*, while the MC-A series samples do not demonstrate such behavior. With the increase of the content of MXene, the resonance peak is “submerged” in the increased dielectric dispersion. For example, in the MC-2 sample, the resonance frequency occurs at approximately 13 GHz. With increasing density, the frequency shifts to higher frequencies at around 15 and 16 GHz. Generally, the dielectric loss is mainly related to the polarization relaxation and conduction loss [29, 64]. The former involves electron polarization, ion polarization, dipole polarization, and interfacial polarization. Therein, the electron polarization and ion polarization are relatively weak in the microwave range, thus the dipole and interfacial polarizations are the major contributors to the dielectric loss at the frequency range of 2–18 GHz. These orientational resonances are mainly due to orientationally induced polarization, while the multiple reflections and scattering as well as structural resonance of EM in the 3D MC cavities may enhance the dipole response [43, 51, 63, 69]. It is noteworthy that while the impedance matching and loss coefficient of MC-3 are lower than MC-4, its absorption intensity is higher. It is implied that this MC oriented cavity structure might possess metamaterial effects [19, 56, 70]. In brief, the optimized spatial distribution and tight binding of MXene and CNF form a three-dimensional continuous conductive network (Fig. S2), which allows efficient absorption through conduction and eddy current losses under the multiple scattering effect of the MC cavity structure. Meanwhile, the transport electrons and tunneling electrons in the conductive network further propagate the induced electric field throughout the three-dimensional structure. As a result, the intercalation and co-assembly of the insulating phase CNF with the conducting phase MXene builds a rich heterogeneous interface. Under the action of alternating electromagnetic fields, these inhomogeneous charges accumulate to form new dipole polarization centers, which cooperate with dielectric polarization to absorb electromagnetic waves [9, 56, 70, 71].

We have further evaluated the effects of permeability. Notably, the negative *μ*″ value denotes that the magnetic energy is radiated out from the induced magnetic field of magnetic materials and transferred into the electric energy (Fig. S3). For non-magnetic MXene, this change in permeability is speculated to the electromagnetic coupling induced by eddy currents. According to the Maxwell's electromagnetic theory, the alternating electric field generates the magnetic field, and the eddy current loss is generated in in the process. According to the C_0_-ƒ curves shown in Fig. S5, in the 4–7 and 10–16 GHz, the overall change of C_0_ is stable, which indicates the widespread eddy current losses, while MC-4A shows additional resonance losses in the 7–10 GHz. According to the ferromagnetic resonance theory and Eqs. S1–S5 [43], the high-frequency magnetic losses may originate from natural resonance and exchange resonance besides eddy currents, and the latter generally occurs in the bands higher than 10 GHz. Therefore, the natural resonance appearing in this band may derive from the unpaired electrons at the transition metal atoms or vacancy defects contained in the MXene lamellae, and these unpaired spin electrons act as net spin units to provide the magnetic moments through the highly anisotropic MC that enables the long-range ordered magnetic coupling. It is known that higher anisotropy coefficient is effective in contributing to the magnetic resonance response.

Anisotropic electromagnetic response brings profound effects on EM absorption. In the comprehensive comparison in Fig. 4d–f, it is observed that different orientations have little effect on the material absorption intensity but significantly impact the central absorption frequency. As density increases, the RL of MC-3A and MC-3R can reach − 50.9 and − 52.6 dB, respectively (Fig. 4g–i). It is important to note that the center absorption frequencies in MC-2A are spaced 1.1 GHz apart from MC-2R. As density increases, the center frequency spacing gradually increases, with a maximum spacing of 7.3 GHz occurring between MC-4A and MC-4R. Additionally, the effective absorption band shifts from the X-band to the S-band.

The unique anisotropic dielectric response characteristics of MXene may be attributed to various factors. Firstly, there may be differences in the effective medium content in different orientations [72]. However, it is obvious that the variation in the effective medium content is insufficient to explain the dramatically changed dielectric constant compared to the higher density samples. Therefore, the anisotropic MC cavities induced changes in the carrier transport properties are one of the important reasons [73, 74]. Due to electromagnetic waves are transverse waves with electric field vectors and magnetic field vectors perpendicular to the direction of propagation. Therefore, the MXene parallel to the direction of the induced electric field ($\overrightarrow{E}$) is theoretically more likely to align with the vector direction of $\overrightarrow{E}$, and effectively inducing carrier transport in the MXene plane as well as dipole polarization and interface polarization, providing greater charge storage and conductivity [44, 46]. In addition, the orientation-derived magnetic resonance coupling of the MXene cavity also profoundly affects the anisotropic electromagnetic response.

**Fig. 5:**
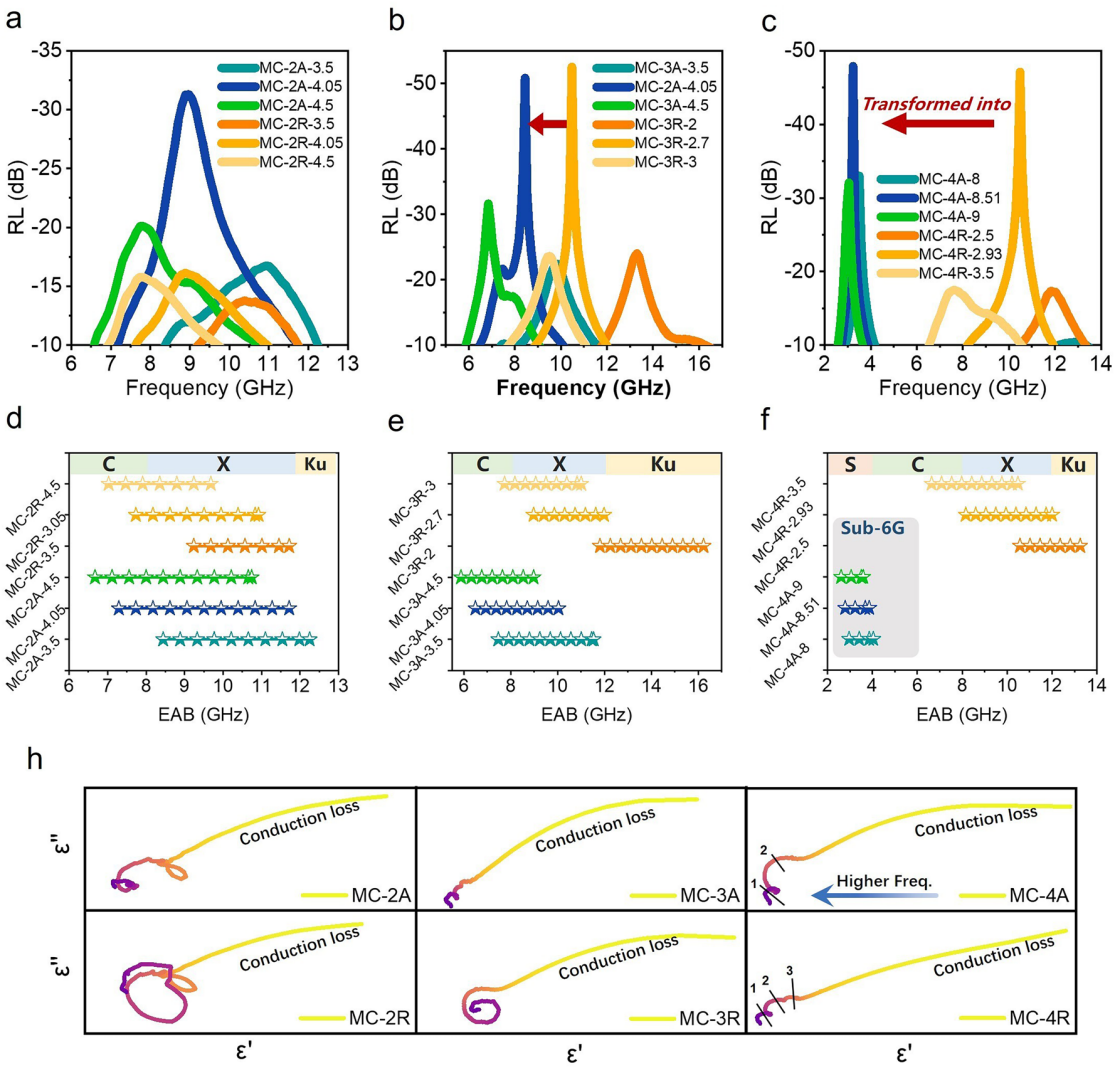
**a**–**c** RL versus frequency of MC-2, MC-3 and MC-4 in different matching thicknesses and **d**–**f** corresponding effective absorption band distributions. **h** Cole–Cole relationship diagram

In 5G communication technology, the Sub-6G band is widely used in the low- and mid-frequency bands, and wireless communication technologies including Wi-Fi, Bluetooth, Zigbee, and LoRa are used to realize low-latency, high-speed, and large-capacity communications. However, low-frequency absorbing materials suitable for this frequency band are very scarce so far. Figure 5a–f illustrates the separation of absorption bands due to differences in material cavities orientation toward the direction of electromagnetic wave propagation. The effective absorption bandwidth (EAB) of the MC-4R moves to 8.2–12 GHz, corresponding to the strongest RL of − 47.9 dB, while the EAB of the MC-4A is located in the lower band of 2.8–3.9 GHz, corresponding to the strongest RL of − 47.1 dB. The absorption band separation becomes more significant at higher MXene contents. Further optimization allows the EAB of the MC-4A to cover the commonly used 5G band (2.5–3.7 GHz) in mobile communications. According to the one-quarter wavelength theory (Eq. S6 and Fig. S6), when the thickness reaches one-fourth of the wavelength of the incident wave, an interference cancellation occurs between the incident wave and the reflected wave, and in this case the thickness is the optimal thickness corresponding to the matching frequency [43, 75]. Figure S5 shows that the measured matching thickness is basically consistent with the simulated thickness of the half-wavelength loss.

The mechanism of polarization loss is further confirmed by the Cole–Cole plots. When the locus of the dielectric constant in the complex plane is a semicircle with endpoints on the axis of reals and center below this axis a polarization type is formed. Below this axis a polarization type is formed. The more obtained circular arcs by drawing *ε*′ versus *ε*″ describe the more polarization types, while the long “trailing” feature represents the occurrence of conduction loss [43, 76–78]. Evidently, there is a competitive relationship between Debye relaxation and conduction loss, and as the conductivity increases, conduction loss dominates the more superior absorption. Meanwhile, by modulating the orientation structure to align the conductive path with the direction of the electric field vector, the type of polarization of MC-R is further enhanced, including interfacial dipole polarization and defect-induced polarization, as well as dielectric polarization generated by CNF (Fig. 5), which is consistent with the occurrence of dielectric resonance of MC-R in the high-frequency region of 12–14 GHz.

### Low-Frequency Performance and Mechanism

**Fig. 6:**
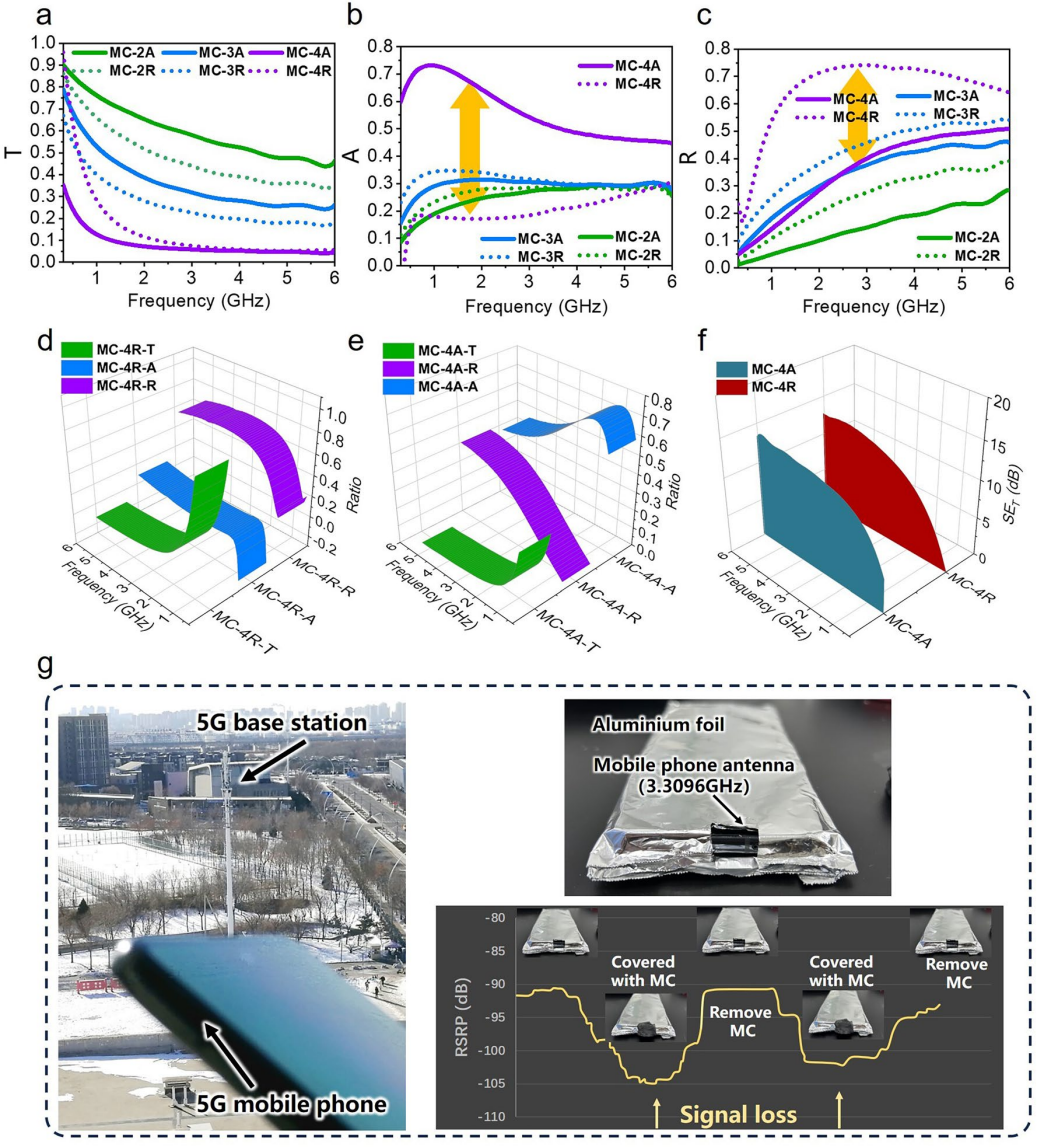
**a**–**c** Absorptivity (*A*), reflectivity (*R*) and transmission (*T*) coefficients of MC at different wavebands. **d, e** Relationship between absorption (*A*), reflection (*R*) and transmission (*T*) coefficients of MC-4 in “MC-R” and “MC-A” orientations. **f** EMI SE of MC-4A and MC-4R. **g** Tests of the effect of MC-4A coverage on the received signal strength of a 5G cell phone

The properties of microwave absorption (MA) and the low-band advantages of the MC cavities were comprehensively analyzed through a comparison of the associated transmission and electromagnetic interference shielding efficiency (EMI SE) in the 1–5 GHz range, as depicted in Fig. 6. When a material is irradiated with microwave radiation, it undergoes reflection, absorption, and transmission. The power coefficients of absorptivity (*A*), reflectivity (*R*), and transmission (*T*) can be calculated from S parameters using Eqs. (5–7). The values of SE_total_, SE_abs_ and SE_ref_, obtained from Eqs. (8) to (10), represent the total EMI SE, the reflection part, and the absorption part of EMI SE, respectively [4].

5 $$R={\left|{S}_{11}\right|}^{2}$$

6 $$T={\left|{S}_{21}\right|}^{2}$$

7 $$A=1-R-T$$

8 $$ {\text{SE}}_{{{\text{ref}}}} \left( {{\text{dB}}} \right) = - 10\log \left( {1 - R} \right) $$

9 $$ {\text{SE}}_{{{\text{abs}}}} \left( {{\text{dB}}} \right) = - 10\log \left( {T/\left( {1 - R} \right)} \right) $$

10 $$ {\text{SE}}_{{{\text{total}}}} \left( {{\text{dB}}} \right) = 10\log \left( {P_{I} /P_{T} } \right) = {\text{SE}}_{{{\text{ref}}}} + {\text{SE}}_{{{\text{abs}}}} $$

The EMI shielding mechanism is primarily attributed to the abundant functional groups and defects, excellent electrical conductivity, high specific surface areas, and numerous interfaces in the material [15, 79]. Figure 6a–c displays the *A*, *T*, and *R* coefficients at 1–5 GHz. The T coefficients of “MC-A” in each sample are notably higher than those of “MC-R”. In MC-4 (Fig. 6b, c), the A-value of MC-4A reaches a maximum of 0.74, surpassing all comparison samples, while the *A*-value of MC-4R is relatively low, around 0.2. Additionally, the average *R*-value of MC-4A is significantly lower at 0.33 compared to the 0.65 of MC-4R, indicating that MC-4A can reduce reflections from surfaces while exhibiting stronger absorption properties. Figure 6d, e provides a 3D projection plot of the *A*, *T*, and *R* coefficients of MC-4 for a more intuitive representation.

Furthermore, the T values of MC-4A and MC-4R were 0.004 and 0.006, respectively, corresponding to SE_total_ values of 22 and 24 dB (Fig. 6f). Although the SE_total_ of MC-4A and MC-4R are similar, the orientation results in distinct shielding patterns, with MC-4A demonstrating strong absorption while MC-4R is characterized by reflective shielding. We also evaluated the effectiveness of MC-4A in shielding signals from cell phones operating in the 5G band. The phone was wrapped in aluminum foil and an antenna receiving hole was exposed for recording Reference Signal Receiving Power values (RSRP). The results show the good shielding effect of MC-4A. (Fig. 6g).

**Fig. 7:**
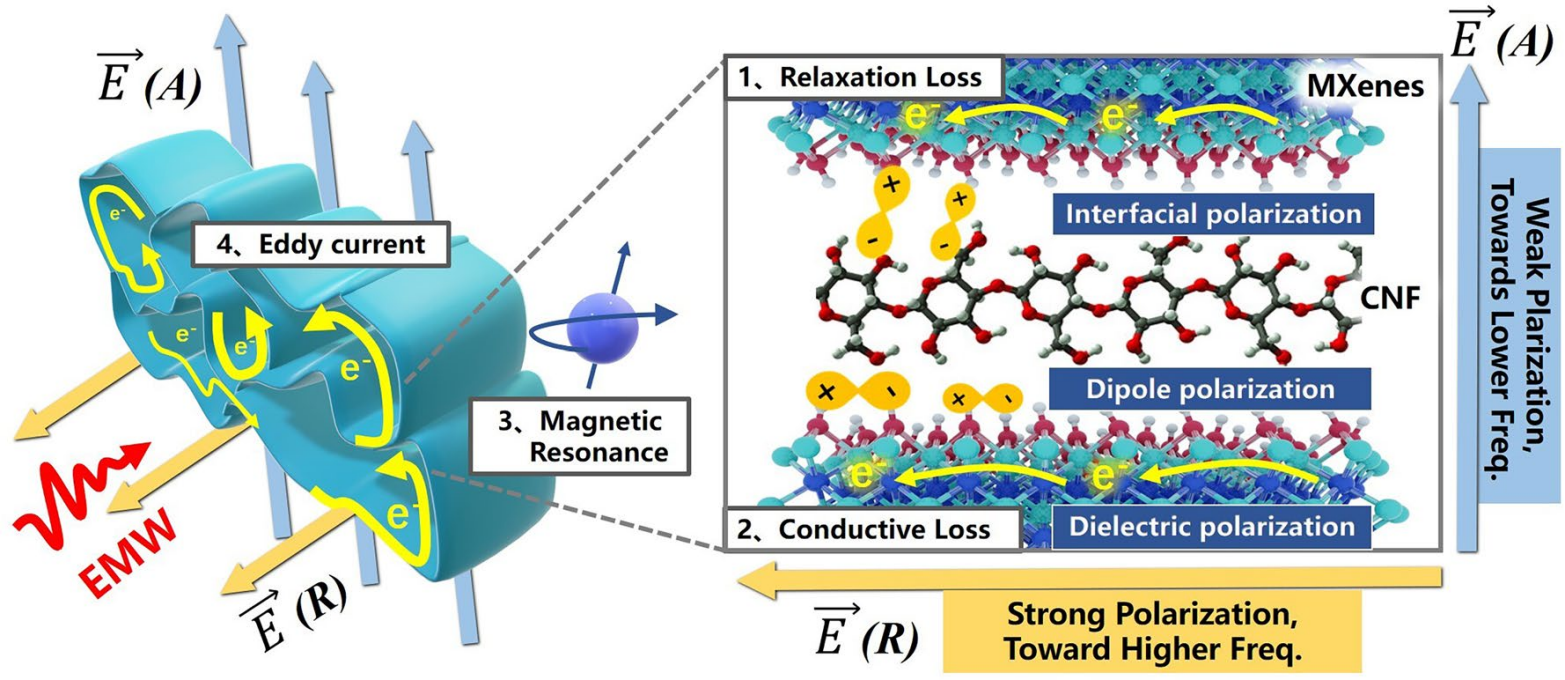
Schematic illustration of the electromagnetic response mechanism of the MC cavities

Figure 7 illustrates the schematic diagram of the response of the 3D oriented cavities structure under electromagnetic irradiation from different orientation. In our study, we developed 3D MXene aerogels characterized by highly aligned cavities, uncovering a straightforward approach to shift the EM absorption band of MXene toward lower frequencies. By adjusting the propagation direction of electromagnetic waves to align with the orientation of the 3D cavity structures, effective electromagnetic coupling is achieved. Furthermore, different orientations lead to varied polarization induction mechanisms and magnetic resonance mechanisms. Specifically, the MC-R orientation, parallel to the direction of the electric field vector, exhibits a richer variety of Debye relaxation types. Conversely, in MC-A, the types of polarization are limited; however, when the 3D cavity structure is perpendicular to the electric field vector, magnetic resonance and eddy current effects are optimized. This is attributed to the long-range ordered magnetic coupling produced by unpaired spin electrons at the sites of transition metal atoms or defects within the highly anisotropic cavity structure of MXene. Therefore, electromagnetic waves interacting with different structural coupling modes derive differentiated networks of conduction loss, eddy current, magnetic resonance, and polarization losses. In summary, we elucidate the distinct electromagnetic response characteristics of MXene/CNF to high- and low-frequency microwaves, paving the way for the application of MXene across a broader frequency spectrum for enhanced wave absorption [5, 11, 13, 15, 37, 39–43, 80–88].

**Fig. 8:**
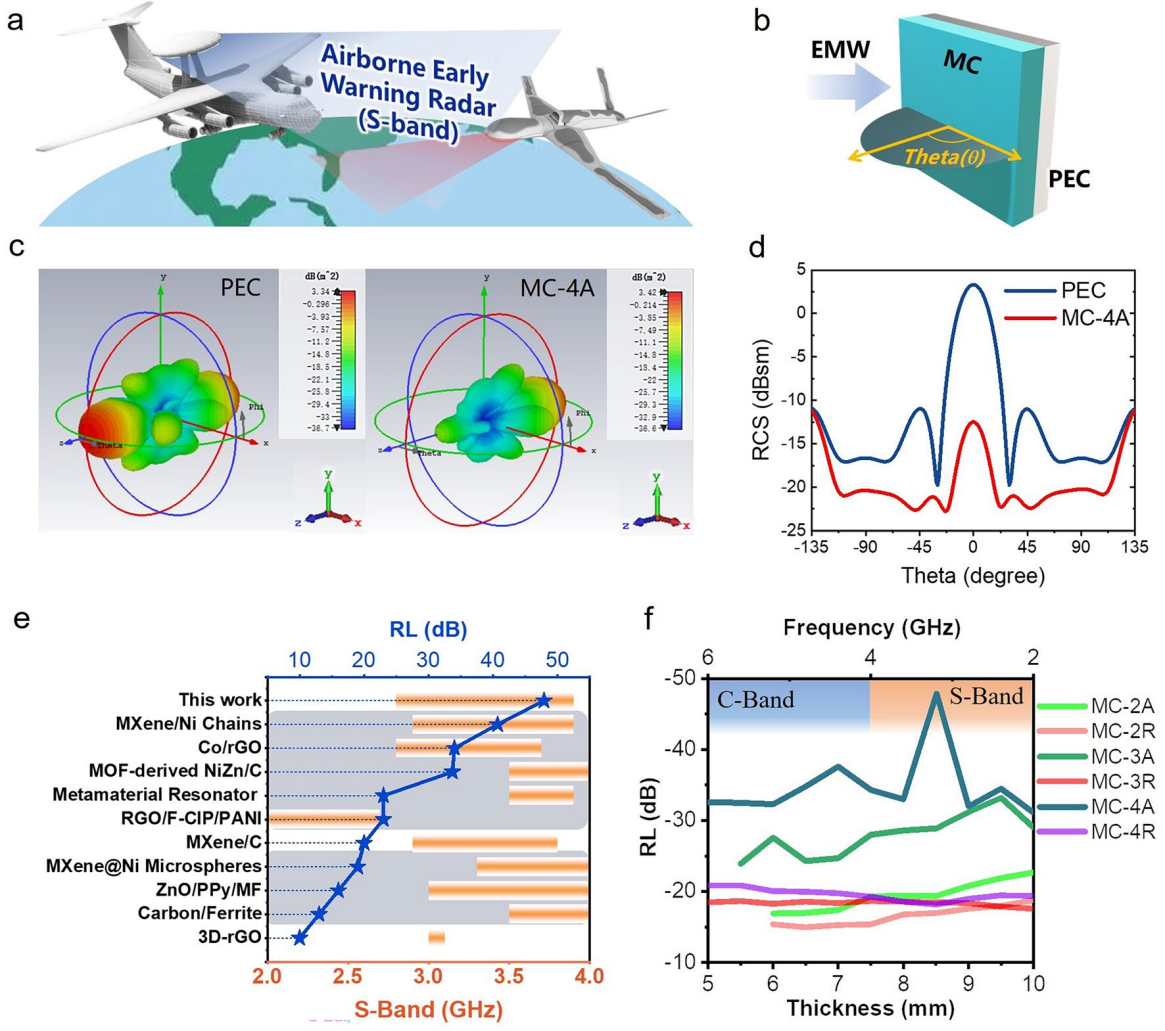
**a** Schematic of airborne early warning radar working in S-band. **b** The model of the absorbing material is set as a square flat plate with a side length of 100 mm, and the bottom is a reflective plate made of PEC with the same side length. **c** Simulation results of CST Studio 2018. **d** RCS of PEC and MC-4A in different azimuth. **e** Comparison between this work and previous low-frequency absorbing materials, where the gray substrate represents that it contains magnetic material. **f** Low-frequency absorption characteristics of the MC

Considering that advanced airborne early warning radars typically operate in the S-band, which allows for long-distance monitoring, many wave-absorbing materials struggle to respond effectively in this frequency range. To address this issue, we conducted a single-station RCS simulation of the MC-4A stealth material in the S-band using CST Studio 2018. The results demonstrate that MC-4A exhibits superior wide-angle stealth performance across an azimuth range of − 135° to 135°. Particularly, under vertical incidence (theta = 0), the material shows a significant reduction in reflection, with the RCS decreasing from 3.3 to − 12.5 dB, reducing the reflection cross-sectional area to only 2.6% of the initial state (Fig. 8a–d). In fact, the low-frequency absorption range of the MC extends to the entire S + C band (Fig. 8e, f). Additionally, the low-frequency absorption range of MC extends throughout the S + C band (Fig. 8e, f). Overall, in the S-band (2–4 GHz), MC-4A showcases strong absorption capabilities and superior low-frequency scalability compared to most carbon-based and magnetic-containing materials [5, 11, 13, 15, 37, 39–43, 80–88].

## Conclusions

In this study, we have successfully constructed anisotropic MC 3D cavity structures with long-range orientation. The ultra-thin pore walls of the cavities have facilitated the oriented stacking of MXene nanosheets, allowing for the controllable tuning of the response of abundant polarization centers such as functional groups, dangling bonds, and heterogeneous interfaces to electromagnetic waves. Our findings indicate that when the MC has an opening direction perpendicular to the electric field direction, the polarization effect induces an electric field that is effectively suppressed, leading to localized conduction losses and enhanced low-frequency absorption. Different orientations of the MC exhibit distinct absorption bands. For instance, in the MC-A orientation, the microwave absorption value in the S-band reaches − 47.9 dB, while the MC-R orientation shows favorable absorption in the X- and Ku-bands, with a maximum absorption intensity of − 52.6 dB. Our study proposes a general strategy for fabricating MXene-based materials for low-frequency tuned absorption in the absence of magnetic element participation, while orientation-induced polarization and the derived magnetic resonance coupling are the key controlling factors of the method. This will open up new avenues for the development of low-frequency, lightweight, and eco-friendly EMW absorption materials.

## Supplementary Information

Below is the link to the electronic supplementary material.

Supplementary file1 (DOCX 740 KB)
