# Supplementary material for: Enhancing Low-Frequency Microwave Absorption Through Structural Polarization Modulation of MXenes
Source: Nanomicro Lett. 2024 Jun 11;16:212. doi: 10.1007/s40820-024-01437-x (PMC11166627; doi:10.1007/s40820-024-01437-x)
Supplement: Supplementary file 1 — Supplementary file1 (DOCX 740 KB) [file 40820_2024_1437_MOESM1_ESM.docx]

Supporting Information for

**Enhancing Low-Frequency Microwave Absorption through Structural Polarization Modulation of MXenes**

Bo Shan^1,2^, Yang Wang^2^, Xinyi Ji^2,^*, Yi Huang^2,^*

^1^ College of Light Industry Science and Engineering, State Key Laboratory of Biobased Fiber Manufacturing Technology, Tianjin University of Science and Technology, Tianjin 300457, P. R. China

^2^ National Institute for Advanced Materials, Tianjin Key Laboratory of Metal and Molecule Based Material Chemistry, Key Laboratory of Functional Polymer Materials, Collaborative Innovation Center of Chemical Science and Engineering (Tianjin), School of Materials Science and Engineering, Nankai University, Tianjin 300350, P. R. China

*Corresponding author. E-mail: [yihuang@nankai.edu.cn](mailto:yihuang@nankai.edu.cn) (Yi Huang); xyji06@nankai.edu.cn (Xinyi Ji)

**Supplementary Figures**


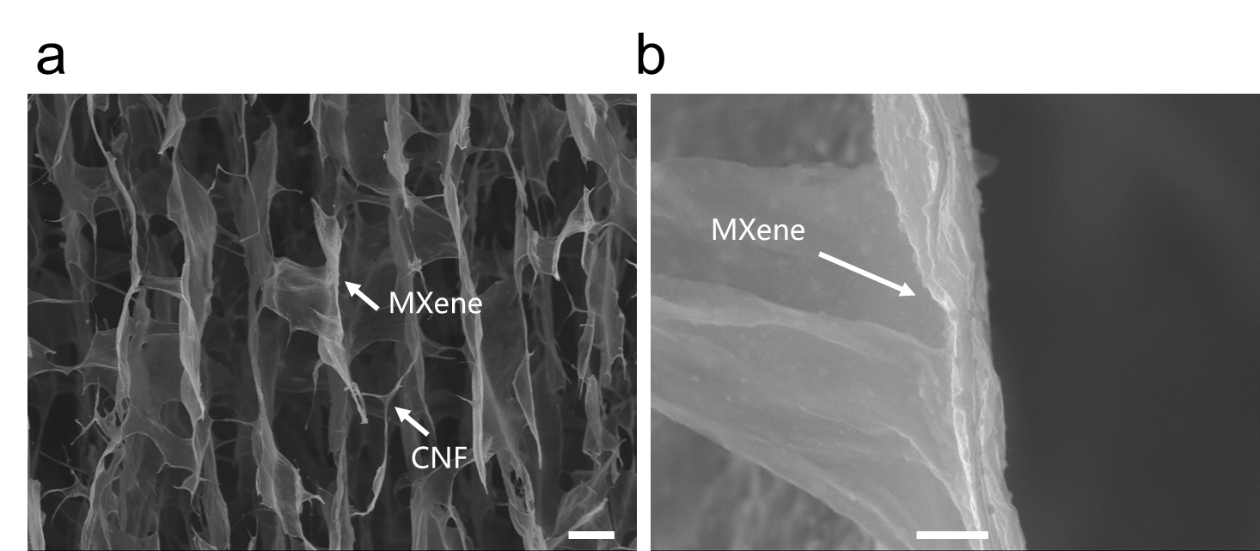


**Fig. S1** SEM images of MC aerogel at different magnifications, the assembled structure of Mxene lamella can be clearly observed, the bar scale is (**a**) 20 μm, (**b**) 1 μm


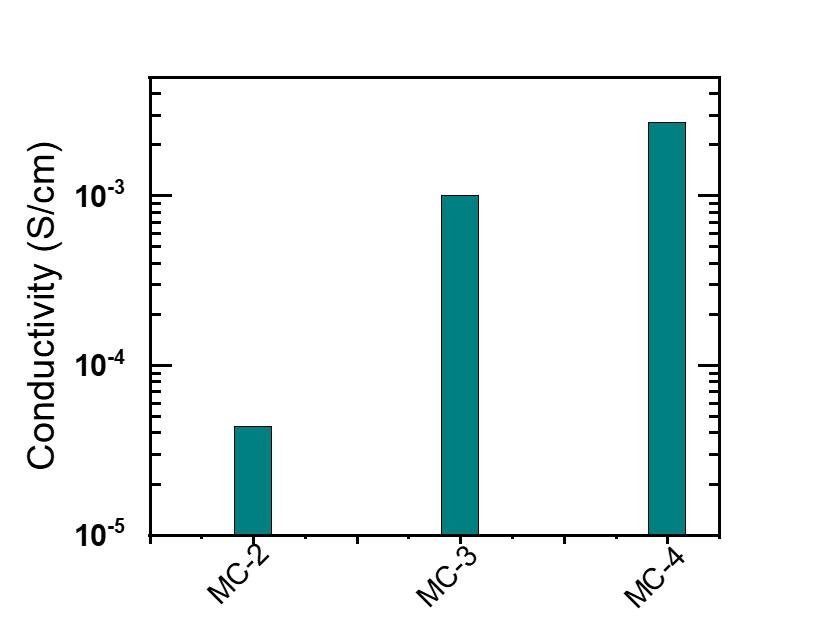


**Fig. S2** Electrical conductivity of different samples


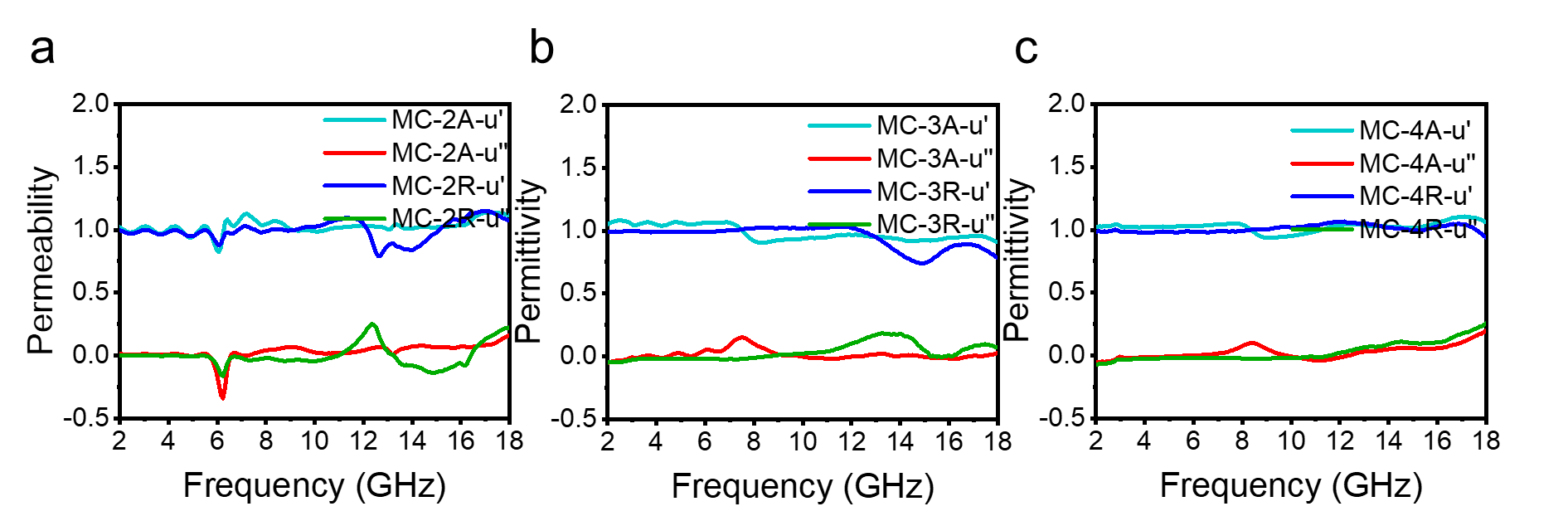


**Fig. S3** Permeability u′ and u′′ as a function of frequency in 2-18 GHz (**a**) MC-2A, MC-2R, (**b**) MC-3A , MC-3R, (**c**) MC-4A, MC-4R


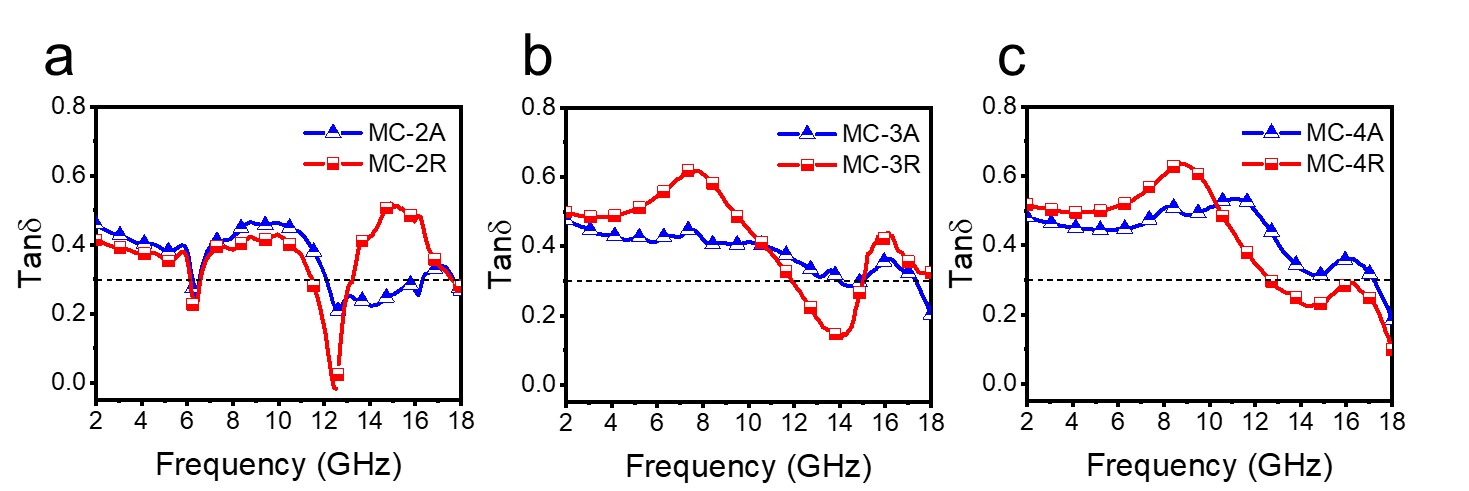


**Fig. S4** Dielectric loss angular tangent versus frequency in 2-18 GHz (**a**) MC-2A, MC-2R, (**b**) MC-3A , MC-3R, (**c**) MC-4A, MC-4R

**Magnetic Loss Factors**

In the high frequency region, the magnetic loss mainly depends on the natural resonance, exchange resonance, eddy current loss, magnetic coupling effect, and so on. Generally, the relationship between the eddy current loss of magnetic materials, the conductivity ($\sigma$) and matching thickness (d) can be expressed as:

$\mu^{''}\approx2\pi\mu_{0}\left( \mu^{'} \right)^{2}\sigmaⅆ^{2}f/3$ (S1)

where $\mu_{0}$ is the vacuum permeability. It can be seen that higher conductivity will result in higher eddy current losses. If the magnetic loss originates only from the eddy current loss, a constant named C_0_ can be obtained by deforming the above equation.

$C_{0}=\mu^{''}\left( \mu^{'} \right)^{-2}f^{-1}=2\pi\mu_{0}\sigmaⅆ^{2}/3$ (S2)

If C_0_ is not a constant, there may be natural resonance losses and exchange resonance losses in addition to eddy current losses in high frequencies. In general, the natural resonance usually occurs at 2-10 GHz, while the exchange resonance mainly occurs above 10 GHz. According to the ferromagnetic resonance theory, the natural resonance can be expressed as:

$2\pi f_{r}=\gamma H_{a}$ (S3)

$H_{a}=4\left| K_{1} \right|{/3\mu}_{0}M_{s}$ (S4)

$K_{1}=\mu_{0}M_{s}H_{c}/2$ (S5)

$f_{r}$is the natural resonance frequency, γ is the gyromagnetic ratio, $H_{a}$ is anisotropy energy and$\left| K_{1} \right|$is the anisotropy coefficient, $M_{s}$ is the saturation magnetization strength.”


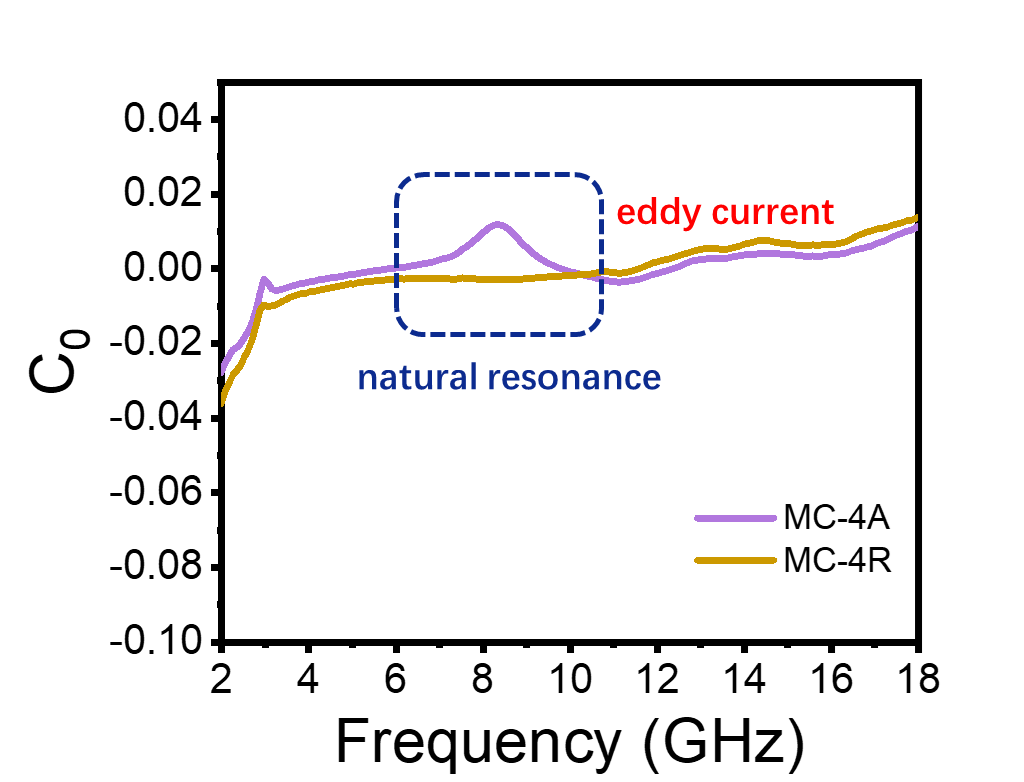


**Fig. S5** C_0_-ƒ curve of MC aerogels


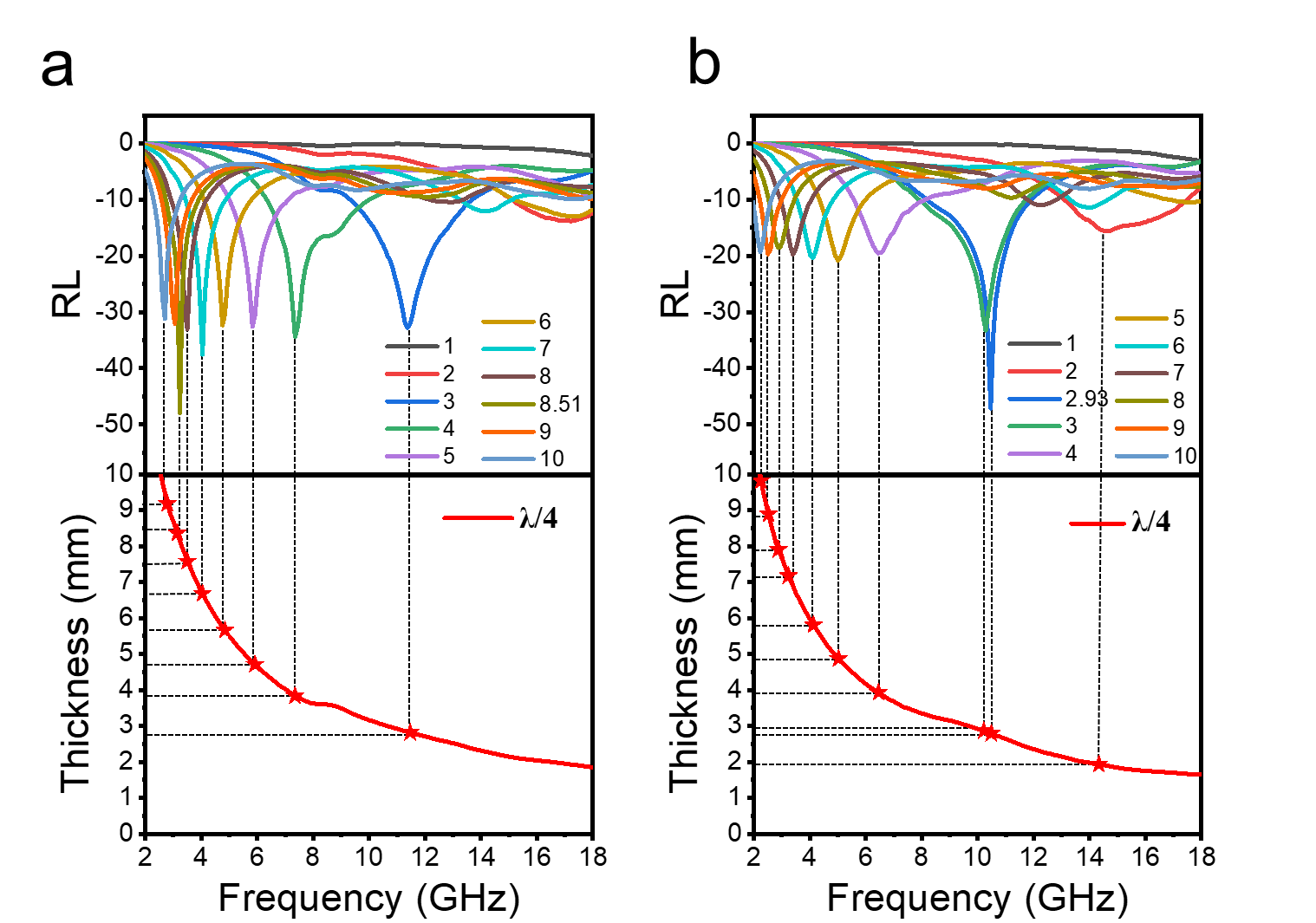


**Fig. S6** RL-f curves and dependence of matching thickness (*t*m) on matching frequency (ƒm) of (a) MC-4A and (b) MC-4R aerogel at the wavelength of 1/4 λ.

$t_{m}=n\frac{\lambda_{e}}{4}=n\frac{c}{4f_{m}\sqrt{\left| \varepsilon_{r} \right|\mu_{r}\mid}} (n=1,3,5\ldots)$ (S6)
